# Rapid Assessment of Stakeholder Concerns about Public Health. An Introduction to a Fast and Inexpensive Approach Applied on Health Concerns about Intensive Animal Production Systems

**DOI:** 10.3390/ijerph14121534

**Published:** 2017-12-11

**Authors:** Marleen Kraaij-Dirkzwager, Joost van der Ree, Erik Lebret

**Affiliations:** 1 Department for Environmental Health, Aftercare and Security, Centre for Environmental Safety and Security (VLH), National Institute for Public Health and the Environment (RIVM), Postbus 1, 3720 BA Bilthoven, The Netherlands; 2 Centre for Sustainability, Environment and Health (DMG) National Institute for Public Health and the Environment (RIVM), Postbus 1, 3720 BA Bilthoven, The Netherlands; joost.van.der.ree@rivm.nl; 3 Chief Science Officer Integrated Risk Assessment, National Institute for Public Health and the Environment (RIVM), Postbus 1, 3720 BA Bilthoven, The Netherlands; Erik.lebret@rivm.nl; 4 Institute of Risk Assessment Sciences, Utrecht University, Yalelaan 2, 3584 CM Utrecht, The Netherlands

**Keywords:** stakeholders, perceptions, public health, environmental hazards, animal husbandry, risk governance

## Abstract

To effectively manage environmental health risks, stakeholders often need to act collectively. Stakeholders vary in their desire to act due to many factors, such as knowledge, risk perception, interests, and worldviews. Understanding their perceptions of the issues at stake is crucial to support the risk governance process. Even though concern assessment is a pivotal element of risk governance, few tools for rapid assessment are reported in the literature. We tested a rapid and relatively cheap approach, taking the Dutch debate on Intensive Animal Production Systems (IAPS) and health as an example. Dutch policy-oriented publications on IAPS and health and ten semi-structured in-depth interviews with a variety of stakeholders were analyzed to identify stakeholders and concerns involved in the Dutch debate about IAPS and health. Concerns were mapped and a stakeholder network was derived. Three classes of concerns were recognized in the discussions about IAPS and health: concerns related to health risks, concerns regarding the activity causing the risks (IAPS), and concerns about the process to control the risks. The notions of ‘trust’ and ‘scientific uncertainty’ appeared as important themes in the discussions. Argumentation based on concerns directly related to health risks, the activity causing the risk (IAPS), and its risk management can easily become muddled up in a societal debate, limiting the development of effective action perspectives. Acknowledging these multiple stakeholder concerns can clarify the positions taken by stakeholders and allow for more and other action perspectives to develop.

## 1. Introduction

Environmental health risks are seldom ‘simple’ risks. Simple risks are risks for which the cause and consequences are known, no large uncertainties limit our knowledge, and most stakeholders agree about cause, effect, and best management strategy. There is little interpretative or normative ambiguity [1]. The traditional approach to risk assessment (chances of occurrence multiplied by impact), risk management, and risk communication is sufficient. However, many environmental health risks can be determined as ‘systemic risks’: multi-dimensional problems operating as part of more wide-ranging physical, social, economic, and political domains, embedded in a high degree of uncertainty and ambiguity [1]. Risk assessment and management of environmental health risks can be challenging due to multiple risk factors, multiple exposures, and multiple health effects embedded in a context of social, economic, legal, and cultural systems. There can be substantial scientific uncertainties (e.g., limited scientific evidence about exposure or health risks) and different views about the main values involved causing ambiguity (e.g., risk assessment and management as part of responsible entrepreneurship versus risk assessment and management through governmental regulations). Environmental health risks can transcend different spatial and administrative scales (from local to transnational), and may be characterized by long delay periods between cause and effect (e.g., the emergence and spread of pollutants, toxic substances, or resistant microbes). “Issues of fairness and environmental justice, visions of future technological developments, and societal change and preferences for desirable lifestyles and community life have a major role in those [systemic] debates” [2].

The framework proposed by the International Risk Governance Council stresses the importance of integrating scientific, economic, social, and cultural aspects of risks in the risk assessment stage. It also proposes the inclusion of stakeholders in the risk assessment and risk management activities [1,2,3]. In The Netherlands, the value of this approach is acknowledged by the major scientific advisory boards: the reports of the National Institute for Public Health and the Environment ‘Dealing sensibly with risks’ (‘Nuchter omgaan met risico’s) [4] and ‘A scan of the safety and quality of our habitat’ (‘Een scan van de veiligheid en kwaliteit van onze leefomgeving’) [5]; ‘Uncertain safety: allocating responsibilities for safety’ (‘Onzekere veiligheid’) from the Scientific Council for Government Policy [6] and the reports ‘Prudent precaution’ (‘Voorzorg met rede’); and ‘Health risks of animal husbandry’ (‘Gezondheidsrisico’s rond veehouderijen’) from the Health Council [7,8]. 

Risk assessment integrating scientific, economic, social, and cultural aspects of risks includes framing the issues at stake and assessment of stakeholder concerns as a basis for deliberate stakeholder involvement [1,3,9,10,11]. Concern assessment and ‘framing the issues at stake’ acknowledges the fact that several problem perspectives and risk attitudes can exist at the same point in time. It is challenging to assess concerns, frames, and risk perception as it entails many factors, such as psychological and cultural factors, desire to influence risks (including application of the precautionary principle), and feelings about distributive justice of risks (fairness) [10,12,13,14,15,16,17]. Scientific literature differentiates between expert and lay risk perceptions. However, in systemic risks (risks that require multi-disciplinary action to assess and mitigate them), it can be difficult to determine who is an expert and who ‘only’ has a lay perspective. Also, scientific experts envision the issues at stake and consequently their cause and action perspectives differently by definition. Scientific uncertainties can easily become the focus with (perceived) scientific controversy triggering further societal debate.

Contrary to the development of methods for technical risk assessment (e.g., multidisciplinary statistical or exposure models), there are yet no validated ‘best’ tools and standard procedures to investigate societal or stakeholder concerns in a debate about environmental health risks. Where different indicators for e.g., disease burden, economic impact, or policy performance exist, indices for perceptions (and acceptability) of risk are currently lacking [18]. This is all the more surprising, considering the recognized influence of stakeholders in crisis management [19] and crisis communication [20,21]. Several frameworks and qualitative techniques can be adapted to particular circumstances to elicit arguments, perceptions, beliefs, emotions, and values of stakeholders [3,13,22,23,24,25,26,27]. The framework of the Health Safety Executive [13,27], further developed by Mansfield et al. [28] has our particular interest, as it assumes that societal concerns appear to be about risk-related issues, but are also frequently driven by other ulterior concerns or interests: concerns with substance-derived origins, value-derived origins, process-derived origins, or stakeholder-derived origins. This meets our day to day observations in the assessment of environmental health risks.

In summary: the development of tools for rapid stakeholder- and concern assessment is important to effectively and efficiently achieve support for (collective) decision-making. This is especially important during crises and societal debates when concerns can become more pronounced and new or dormant stakeholders can become very active in mobilizing particular interests. Therefore, the aim of this project is to explore stakeholder concerns in an ambiguous societal debate using a rapid and relatively low-budget approach. 

We took the Dutch debate about the health risks of Intensive Animal Production Systems (IAPS) as, a perfect example of a systemic risk for which stakeholder- and concern assessment is highly recommended [1].

Scientific evidence on the relationship between IAPS and health in The Netherlands recognizes potential health effects of (resistant) microbes (causing outbreaks, complicated infections and depletion of the arsenal of effective antimicrobial drugs), gaseous and particulate pollutants including endotoxines (related to respectively exacerbations of asthma and chronic obstructive pulmonary disease, allergies, and airway hyperreactivity), pollution of the local environment, odour, and psychological effects of living in an industrialized area [29,30,31,32,33]. The relative impact of IAPS on health in The Netherlands in comparison with other environmental health risks (living along highways, industrial areas, etc.) is unknown. The relevance of international studies is limited due to differences in husbandry systems and the exceptionally high combined density of animals and people in The Netherlands.

Many stakeholders, acting at local, national, and international levels, are involved in the societal discussion about the future of IAPS. The Netherlands has a long-standing tradition of animal farming. Many have economically benefited from animal husbandry, and farming has contributed to the development of the Dutch landscape. Current economic incentives drive the concentration of animals and farms, sometimes leading to so-called megafarms, with, for example in The Netherlands, >200,000 chickens or hens or >7500 hogs or holdings. These farms develop amidst communities. The societal debate is fed by the feeling of inhabitants in these communities that they have little influence on these developments. A large Q-fever outbreak (2007–2011) and the emergence of pathogens resistant to antibiotics with animal origin have accelerated and heated the debate. The Q-fever outbreak (2007–2011) caused more than 4000 reported patients. Over 280 patients developed chronic Q-fever and 20% of the reported patients suffer from Q-fever Fatigue Syndrome. Over 70 patients died, probably due to Q-fever-related complications [34,35,36]. Over 50,000 pregnant dairy goats were culled. A regional multi-disciplinary stakeholder platform and a patient-organization were established. Involved costs in the outbreak exceeded at least 161–336 million euro [37,38]. Governance of the Q-fever outbreak was controversial [39,40,41].

Since the Q-fever outbreak, experts and lay-people publicly attribute several health risks to IAPS. Risk managers face the challenge to decide on balanced regulations and possible interventions to limit health risks, while maintaining the economic viability of the agro-food complex for the economy and employment opportunities. For example: in 2009 the agro-food complex contributed 9.9% to the Dutch economy (50.7 billion euro) and 10.2% to national employment [42].

What some see as a problem, others see as a solution, indicating the complexity and ambiguity in the debate; e.g., over-use of antibiotics stimulating the emergence of resistant microbes versus safe-use to prevent zoonotic diseases [43,44,45]. Interventions to decrease health risks and increase health benefits can conflict with other values that are perceived as equally important to human health. For example: a large industrial farm with a closed system of breeding–producing–slaughtering in an industrial area could carry very little public health risks [30]. It might, however, meet little understanding from those advocating animal welfare, defined as ‘allowing animals natural behavior’ [46]. Some promote alternative (small-scale) husbandry systems where animals and humans are in closer contact. However, this ‘biological or traditional farming’ might involve more public health risks, for example due to the potentially increased presence and transmission of zoonotic pathogens [30]. These risks might be better accepted by some, because an alternative farming system would be more in line with other personal values related to animal welfare, spatial planning, and perceived sustainability. There was limited literature on societal concerns and risk perception related to animal husbandry in The Netherlands at the start of the project [47,48,49,50,51].

## 2. Materials and Methods

Stakeholder concerns regarding the development of IAPS were explored using two data sources: (1) analysis of publicly available policy-oriented documents, and (2) interviewing selected stakeholders involved in discussions about IAPS and risk governance. Following Brugha and Varvasovszky [52], Bryson [53], and Reed et al. [54], we defined stakeholders in the societal debate on IAPS as: ‘every person or organization who/which can influence discussions about the future of IAPS in The Netherlands for example through research, policy development, or mobilization of others’.

### 2.1. Assessing Stakeholder Concerns in Policy-Oriented Documents and Semi Structured Interviews

Documents published in the period January 2008–July 2011 were sought through Google scholar using the keywords ‘intensieve veehouderij en volksgezondheid’ (intensive animal husbandry and public health) and ‘megastallen’ (megafarms). Additional documents were identified using ‘snow-ball sampling’ and recommendations from interviewees [29,30,31,32,33,43,44,45,46,55,56,57]. Finally, two letters from the Ministries of Health and Agriculture to the Parliament were selected [58,59] and a transcript of the parliamentary debate on IAPS and health [60], as these political documents reflect societal perceptions and value judgments. 

Semi-structured interviews with a purposive sample of stakeholders were conducted to better understand the argumentation, values, norms, and emotions involved in the debate on IAPS in The Netherlands. The IRGC differentiates four classes of stakeholders: political actors, business actors, scientific actors, and civil society groups. Fourteen stakeholders within these four categories were identified, based on their familiarity with the debate about IAPS and health in The Netherlands: members of national advisory boards on the future of IAPS, authors of policy reports relating to IAPS and/or expressing opinions in the political debates on IAPS, and experts in risk governance of environmental health risks. Ten of the 14 identified stakeholders agreed to participate: three professionals involved in public health management at the local and national level, four academics involved in different Dutch national advisory boards on public health and safety (veterinary medicine, environmental epidemiology, general practice, environmental policy), one representative of the agricultural industrial sector, one policy officer at provincial level, and one politician at national level. Four interviewees were also co-authors of policy documents included in the document analysis. Three selected stakeholders refused due to time-constraints and one stakeholder did not respond to the invitation. A semi-structured topic list explored: (1) the perception of the debate on IAPS and health, (2) argumentation, values, norms, emotions, and interests involved in the debate on IAPS and health, (3) thoughts on ‘the way forward in the discussions/risk governance’, (4) perceived responsibilities in the risk governance process, and (5) control question: personal views and feelings towards IAPS. The one-hour interviews took place in May–August 2011, were performed by one interviewer (first author), audio-taped, and documented with (Dutch) transcripts. Interviewees signed an informed consent form. Maximum care has been taken during analysis and reporting to keep confidentiality for the participants. 

### 2.2. Data Analysis

Analysis of the documents and interview transcripts followed the principles of the Grounded Theory approach [61] and focused on the identification of stakeholders, argumentations, overarching themes, and selection of qualitative text quotes from interviews as narrative reflection of key elements in the debate. The different themes were listed (see Table S1 in Supplementary Materials) and subsequently assessed using the HSE framework [13,27] and the Mansfield framework [28]. The interview transcripts were read again by the interviewer and an independent sociologist, specifically looking for citations grounding or contradicting the identified themes. This coding process, also assuring reliability of the coders through double review of the full transcripts, provided a high degree of consensus on the inducing themes. The first five or six interviews consequently revealed new stakeholders, concerns, arguments, and values involved in the debate. The ensuing interviews confirmed the variety of concerns, arguments, and values involved without revealing unexpected new stakeholders. At this point the researchers collaboratively concluded that saturation was achieved and the main findings were summarized.

## 3. Results

**Figure 1:**
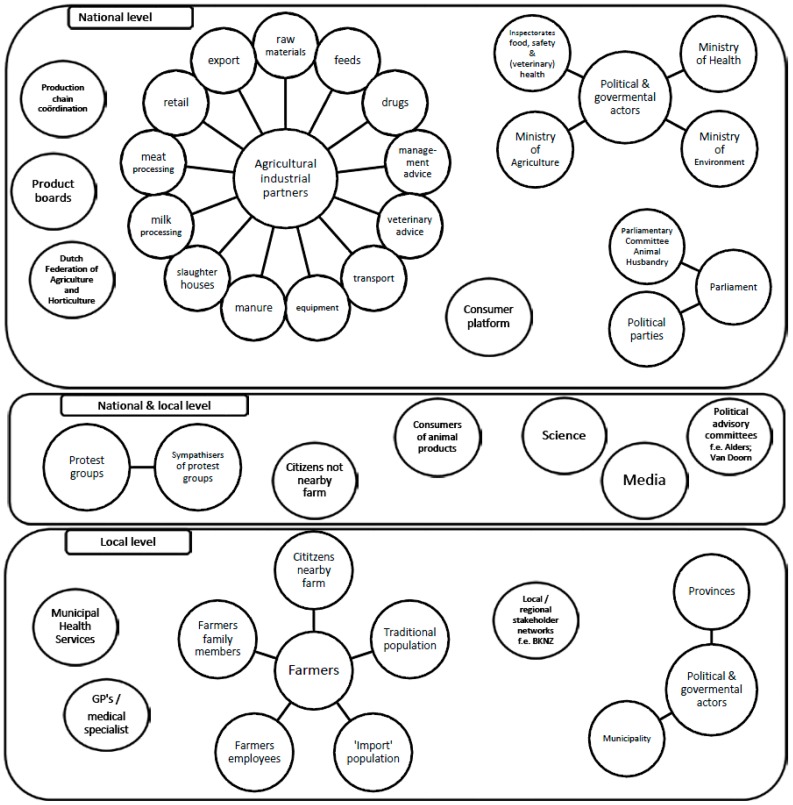
Stakeholders involved in Dutch debate on Intensive Animal Production Systems (IAPS) and health.

A large number of stakeholders were identified, summarized in Figure 1. 

**Figure 2:**
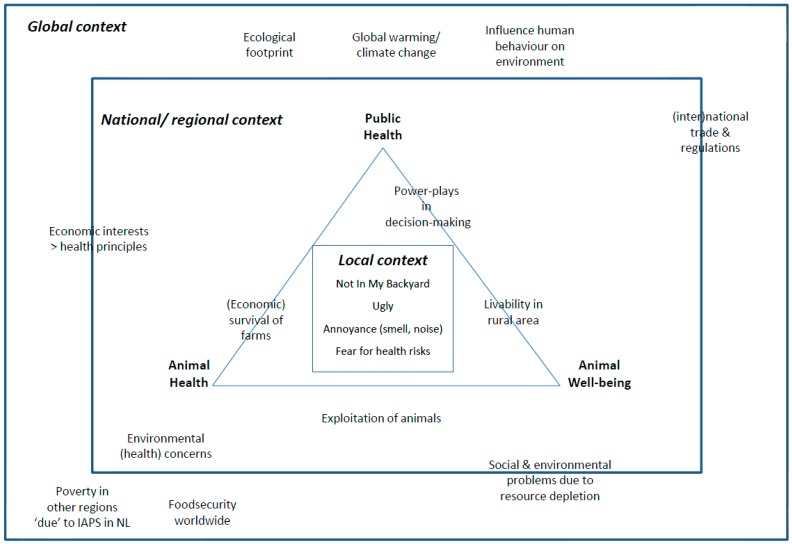
Scaling of societal concerns related to IAPS.

It shows a crowded landscape of stakeholders with varying responsibilities and interests. They interact on different administrative levels and on different issues (Figure 2), causing a complex and dynamic set of arenas for debates.

The document analysis and interview transcripts show the complexity of the discussions about IAPS and health due to the amount of interrelated issues, perceptions, and values involved. The debate on IAPS and health has been characterized by the interviewees as “scattered”, “diffuse and amorphous”, and “better if had taken place at the start of the expansion of IAPS”, but “not bad as all the concepts are getting on the table by now”, even though “it is unclear what we know and which choices we can make”. 

**Figure 3:**
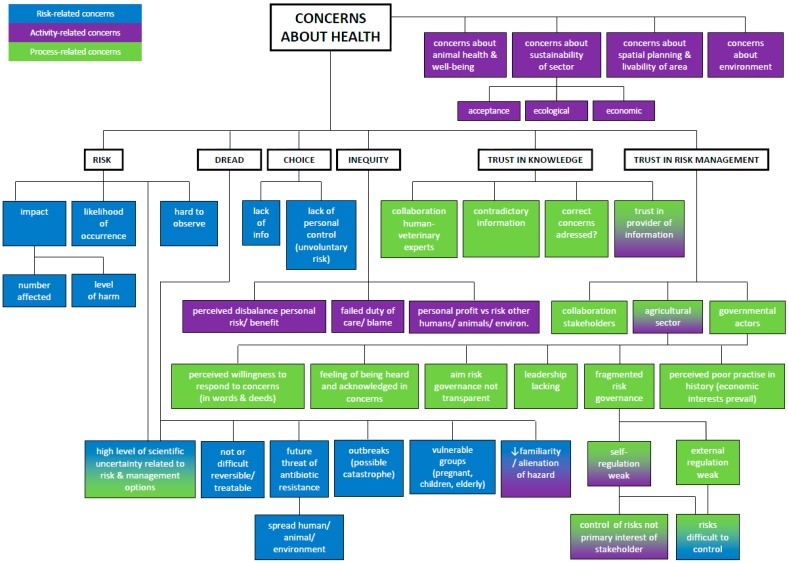
Structured map of stakeholder concerns.

The structured map (Figure 3) summarizes the stakeholder concerns derived from the documents and interviews. It combines the known determinants of risk perception (likelihood of occurrence, impact, trust, dread, choice, inequity) and specific drivers influencing the perception about IAPS. Among these are: the perceived lack of collaboration among risk governors, contradictory information, lacking leadership in complex discussions, weak self-regulation, weak external regulation of the agricultural sector, perceived poor practice in history (economic interests prevail), willingness of risk governors to respond to stakeholder concerns, and unclear (aims of the) risk governance process. We concluded three over-arching themes (recognizable by different colors in Figure 3), believing that acknowledgement of these themes in the discussions can potentially support (public health) policy development:

(I) Concerns about health risks related to IAPS (risks);
(II) Concerns about the industrial agricultural sector (activity causing risks);
(III) Concerns about the management of health risks related to IAPS (process of risk management).

I. Concerns about health risks related to IAPS (blue in Figure 3).

The most frequently mentioned health risks were zoonotic diseases and fine particles in the air (current risks), rapid emergence of pathogens resistant to antibiotics (future risk), and sensitivity to chemical substances, bad odor and sleep disturbance due to noise and light. Interviewees emphasized the high degree of scientific uncertainty related to risks originating from different forms of IAPS (e.g., visibility of the risk, likelihood of occurrence, level of exposure, impact, vulnerable groups) and applicable risk reduction options. The precautionary principle was frequently mentioned as the main argument to take additional control measures in the absence of scientific evidence. Most interviewees recognized that other concerns (e.g., about animals or spatial planning) played a role in the perception of health risks.

“You should gain knowledge, not only led by emotions like a large farm is always bad, a small farm is all ways good, biological farming is always good and bio-industry is always bad. It’s an important question: what is good or bad?”

(Public health advisor at national level)

“The discussions are so entangled, that only a broad answer [addressing all concerns] will be convincing.”

(National politician)

II. Concerns related to agricultural industry (purple in Figure 3).

Many concerns expressed in the discussions about IAPS and health are in fact concerns about the Dutch agricultural industry and its relation with animal health, animal welfare, landscape infrastructure, livability of rural areas, the high density of animals and humans, economic sustainability, concerns about environment, ecological sustainability, and ethical concerns, as listed in Table S1 in the Supplementary Materials. Fewer people are engaged in and dependent on agriculture in The Netherlands than before. They are less in contact with animals and, if so, are mainly on small-scale farms for recreational purposes. The appreciation for the agricultural industry declines and the alienation of agricultural practices and products increases. This can also influence the perception of the large-scale agricultural industry in The Netherlands.

“…for local residents it is ‘I don’t want to live next to an enormous farm’, walls at which I am suddenly staring, all the transportation of manure, soy…it’s about the character of the landscape and whether the Dutch rural areas should be allowed to further urbanize, industrialize.”

(Environmental Epidemiologist- advisor at national level)

“…it’s changed with large industrial complexes for which people have no positive feelings, none at all. People don’t see the farmer any longer, only experience irritation … It leads to conflicts.”

(Public health advisor at local level)

Interviewees also addressed the reputation of the industrial agricultural sector influencing the risk-perception. The industrial sector is perceived as economically driven and characterized by large closed farms, decreased animal well-being, and ruining of the landscape. It contradicts the romantic view of ‘cows grazing green grass in the fields’.

“If there would be a health problem related to the biological animal husbandry, that wouldn’t immediately mean that we don’t want it anymore…we love it a little so to say…but big farms we don’t like at all, it’s industrialized…not transparent.”

(Veterinary health advisor national level)

“If people believe that you [farmer] are inaccurate with your animals, they will also have the idea you are not so concerned with the public health.”

(National politician)

III. Concerns about the management of health risks related to IAPS (green in Figure 3).

We recognized a third class of concerns expressed in the interviews, not directly related to health risks nor the agricultural industry, but the governance process. Interviewees were unclear about the responsibilities related to the management of health risks of IAPS. This includes initiating research, taking precautionary measures if necessary, and developing policy around IAPS for the longer term. This is surprising, as one would expect from key informants, involved in many of these discussions, to be sufficiently aware. They, however, expressed uncertainty about the responsible actors (government or industrial partners) and the main values for policy-making (human-, veterinary- and environmental health, economic risks and benefits). It appears to be unclear who is currently taking the lead in the discussions and policy development.

“We can structure the animal husbandry exactly as we want in relation to [animal] well-being, health risks, risk-acceptability…but you have to make choices… it is important to speak with all partners involved in IAPS…supermarkets, industrial chain coordinators, milk factories, all multinationals…it’s complex as we live in an open international society…it is only possible if government is also involved in these discussions.”

(Veterinary health advisor at national level)

“Government will be addressed [in the discussions] and should have a state of the art story. It should emanate expertise and understanding; have an ear for the problems perceived in society. This combination is not easy, but I think it has high priority. Because it creates trust and this strongly influences the risk perception…. This does not only have to do with communication, but whether you have organized things trustworthy.”

(Public health advisor at national level)

Even though it was not the initial aim of our study, we recognized the reoccurrence of the concepts of ‘uncertainty’ and ‘trust’ within the three overarching categories, addressed by most interviewees. Table 1 therefore summarizes the main classes of concerns (health risks, activity causing the risks and the risk governance process) and the questions to be addressed, focusing on decreasing the uncertainties and increasing trust. 

The interviewees suggested several activities to decrease uncertainty and increase trust among stakeholders. One common suggestion stands out: a shared research and policy agenda for (risk governance of) health risks related to IAPS, developed and presented by all governmental and industrial agricultural stakeholders involved. This suggestion not only emphasizes the desire for more research into health risks, but also explicit leadership and clarity around responsibilities in the complex discussions. 

## 4. Discussion

Our aim was to apply insights on stakeholder concern assessment—as an essential component of risk assessment—to a dynamic and complex societal debate in which ‘human health concerns’ are frequently voiced. At the time of study, there was limited scientific evidence on the relationship between IAPS and health in The Netherlands [8,29,32] and these results themselves became subject of debate [60,62,63]. In such a politicized context, the importance of adequate framing of the ‘issues at stake’ becomes even more salient; ‘public health risks’ can easily become the ‘shared argument’ among stakeholders while covering other primary concerns. We explored the stakeholder concerns involved in the societal discussions about IAPS and health in The Netherlands, a discussion also ongoing in other countries [64,65,66,67]. Using a rapid and relatively low-cost approach, we identified a large array of stakeholders and concerns related to human-, animal- and environmental health, (economic) sustainability, spatial planning, and the risk assessment and risk management process. The variety of concerns, arguments, values, and stakeholders involved in the discussions about IAPS and health shows that IAPS in The Netherlands should be approached as a systemic risk [1] with appropriate inclusive risk assessment and management processes. There are risks that can lead to loss of health and there are concerns that can lead to loss of wellbeing, which the World Health Organization among others includes in its definition of health. Some concerns appear to be fed by a lack of trust in government institutions and the agricultural sector and a lack of clarity about the risk governance process. This leads to people voicing ‘health’ concerns in order to reclaim more influence over their living environment. If unaddressed and of main interest for powerful stakeholders, these underlying concerns can become implicit obstacles in risk assessment, taking away the attention from the development and implementation of risk reduction strategies. Explicit acknowledgement of the varying concerns can support constructive dialogue, including in depth exploration of action perspectives.

### 4.1. Advantages and Disadvantages of Our Approach

A low-cost approach using publicly available policy- and political documents and interviewing a small but varied sample of stakeholders can support initial identification of stakeholder concerns. We recognize the risk of interviewing a small number of interviewees (risk of selection bias) and acknowledge that interviewees provide only revealed truth, i.e., the information that the interviewee is willing to share and ‘personal construct’: the situation seen from the point of the interviewee. Stakeholders will attach different weights to different concerns. The initial stakeholder network (Figure 1) calls for further analysis, including the perceptions, primary and secondary interests, political tactics, and impact of stakeholders. Such an analysis was beyond the scope of this project but is relevant for future public-health decision-making. We also acknowledge the fact that inclusion of societal concerns in the risk assessment process has advantages and disadvantages [17,27,68,69]. Explicitly addressing stakeholder concerns, in some cases through involvement of stakeholders, in the risk governance process can possibly prevent high costs from regulations, unnecessary research, and political damage. Through constructive dialogue, it is supposed to support the development of action perspectives and increase trust among stakeholders. On the other hand, it takes time and integrating public perceptions in risk assessment and management should be done with caution, to avoid (perceived or unwanted) manipulation of the perception or the process. 

### 4.2. Developments after Our Study

Our study was a ‘snapshot’ of the debate at that time. Several developments after completing our study support and strengthen our results. 

In 2013, two years after our study, following stakeholder deliberations [70], the Ministries of Health and Agriculture invested in multi-disciplinary research into the health effects related to IAPS and initiated the establishment of a national Knowledge Platform on Animal Husbandry and Human Health [71]. The Platform consists of seven organizations with a shared aim to ‘collect, assess, appraise, and disclose knowledge about animal husbandry and human- and veterinary health’ in State of Knowledge papers. New scientific evidence related to health effects of intensive animal husbandry is discussed in this platform and made publicly available in language understandable for non-scientific professionals, to help these professionals address questions and concerns of citizens, farmers and entrepreneurs, and politicians. The first series of State of Knowledge papers focused mainly on the health risks and the activity causing the risks. Professionals welcomed the papers but also commented that they lacked risk management and action perspectives. Professionals can address their questions to the platform, of which the secretariat is positioned at the National Institute for Public Health and the Environment (RIVM). These questions focus on the same three overarching themes: concerns about the health risks related to IAPS (risks), the industrial agricultural sector (activity causing risks), and how to manage the health risks related to IAPS (process of risk management). 

Members of the Platform are occasionally invited to local stakeholder meetings in regions where discussions about the expansion of farms or the establishment of manure processors are ongoing between inhabitants, farmers, municipal health services, and representatives of local governments. The goal of the Platform in these cases is to create comprehensive questions and answers (Q & A’s) that connect to the questions and concerns in these local discussions. These Q & A’s are then discussed with the local stakeholders to improve them so that they can be used in equal local settings elsewhere. They observe a shift in themes during the meetings. At first, the discussions concentrate on the health risks and the activity causing the risks and what the available knowledge means for the stakeholders. After a while, without necessarily agreeing on the new development, the discussions shift to how to manage the (concerns about) health risks and how to give inhabitants a sense of control over their own environment. The patterns in questions addressed to the Platform and the observations during regional stakeholder meetings are in line with the classes of concerns which we identified in our study. Other researchers have also recognized the array of frames in the societal discussions and the need to scope the discussions in the different policy arena’s [72]. Research to better understand stakeholder perceptions to support constructive dialogues has recently been initiated [73]. 

### 4.3. Are the Three Overarching Themes Relevant for Other Environmental Health Risks?

Conclusions drawn from a small study like ours are exploratory in nature. However, from the literature on anthropogenic environmental health risks and our operational experience as public health advisors, we recognize the three over-arching classes of concerns: concerns about the health risks, concerns about the activity causing the risks, and concerns about the risk management process. We also recognize the triggering role of ‘uncertainty’ and ‘trust’ in the social amplification of risk. They appear to be influential drivers in societal debates about varying environmental hazards and public health risks (e.g., new emerging chemicals, low frequency sounds from wind turbines, electromagnetic fields). Trust and uncertainty are known to influence risk perception [12]. Uncertainty appears to be related to the lack and interpretation of scientific evidence, but also to the activity causing the risk (often industrial practices with economic benefits) and the risk governance process (often dispersed among public and private actors). Trust also appears to relate to the visibility and transparency of the activity causing the risk and of the risk governors, their responsiveness to societal concerns, their perceived ulterior motives, and their expected influence on emerging events. ‘Trust in response’ not only depends on trust in the risk managers, but also on the preferred action perspectives in response to uncertain risk problems [14,74,75]. The latter relates to the different expectations stakeholders have about the possible and necessary influence to decrease environmental and health risks related to IAPS, including feelings of ‘blame’ and ‘shame’ (accountability) [15,76]. The alienation from agricultural activities and animal products does not help to increase trust in the industrial partners. As Scholz and Siegrist state: “In the absence of sufficient knowledge, trust and affect are important heuristics that guide our decisions” [77]. The policy officer at provincial level also articulated this notion clearly:

That’s the challenge for the industrial agricultural sector, to work hard and get the trust back…people need to trust that they have enough attention for public health, animal welfare and environment…if only two or three free-riders [who do not follow rules/agreements for the sake of their own interests], a few newspaper articles and you can start all over again.

(Policy officer at provincial level)

## 5. Conclusions

To effectively assess and manage environmental health risks, stakeholders often need to act collectively. Stakeholders vary in their desire to act due to many factors, such as knowledge, risk perception, interests, and worldviews. Identifying stakeholders and the ‘issues at stake’ are essential components of integrated risk assessment, recommended for the risk governance of systemic risks like IAPS. We presented our rapid and relatively low-cost approach based on analysis of publicly available policy- and political documents and a small number of interviews as an initial step in understanding the stakeholders and their concerns. It appears to be useful for the governance of environmental health risks (at least those related to IAPS) to differentiate and acknowledge the concerns related to the actual health risks, concerns about the activity causing the risks, and concerns about the risk governance process. Specific attention is needed for the concepts of ‘trust’ and ‘(scientific) uncertainty’, which appear to play a role in each of the three aforementioned themes and are known triggers in societal debates. We argued that our findings, in particular with respect to the structuring and untangling of different kinds of concerns, are relevant to similar debates about environmental health risks in The Netherlands and other countries.

It will remain a challenge for environmental and public health risk assessors to maintain the trust of stakeholders through sound risk assessments and effective science—and risk communication. Our approach to stakeholder- and concern assessment can support environmental and public health risk assessors in two ways. Firstly, to initiate the necessary stakeholder deliberations by creating a shared understanding of the variety of issues at stake and through definition of shared ambition(s) for collaboration. Secondly, the issues summarized in Table 1, can guide experts—often focused on specific risks—to also pay attention to the overarching themes in order to develop and implement effective action perspectives. This widening scope can contribute to drafting a comprehensive research agenda decreasing uncertainties, to exploration of action perspectives including the development of innovative risk reduction strategies and support risk communication by targeting the messages to the main stakeholders, their concerns and information needs. 

Integrated risk assessment and subsequently risk governance of environmental health risks can be further developed through:

(1) Application and evaluation of rapid methodologies to recognize stakeholders and concerns in societal debates. In addition to the approach reported in this paper, this can for example be done by analyzing questions posed to (public) health services and knowledge platforms, discourse analysis of publications and stakeholder dialogues, quick scan surveys, and (social) media analysis; and
(2) Understanding the determinants of ‘trust among stakeholders’, ‘trust in information’, and ‘trust in response’; and
(3) Application and evaluation of analytic–deliberative methods for stakeholder involvement in environmental health risks, which are often characterized by large actor networks and thus a rich variety of knowledge, perceptions, concerns, interests, worldviews, and preferred action perspectives.

## Figures and Tables

**Table 1:** Differentiation of concerns involved in the debate on IAPS and health.

	Uncertainty/Lack of Clarity	Trust
**Concerns about health risks related to IAPS (risk)**	Which risks? How large are the risks? Which options to decrease risks? Now and in the future.	Is there (easy access to) trustworthy and sufficient information on risk and control options?
**Concerns about industrial agricultural sector (activity causing risk)**	Alienation from animal husbandry; (perceived) lack of transparency and visibility	(How) do industrial agricultural partners and/or governmental actors put public health interests before economic interests? Image/visibility of partners
**Concerns about risk management (process to manage risk)**	Who is responsible for (which part of) the risk management? How is responsibility taken? Aim of risk management?	Does the right actor feel responsible and show responsibility? Transparency/visibility of risk governance process.

## Supplementary Materials

The following are available online at www.mdpi.com/1660-4601/14/12/1534/s1, Table S1: Concerns and Values Involved in Discussions about Intensive Animal Production Systems in The Netherlands.

## References

[1] Renn O. (2005). White Paper on Risk Governance—Towards and Intergrative Approach.

[2] Renn O., Klinke A. (2004). Systemic risks: A new challenge for risk management. EMBO Rep..

[3] Renn O., Klinke A., Asselt M. (2011). Coping with Complexity, Uncertainty and Ambiguity in Risk Governance: A Synthesis. Ambio.

[4] RIVM (2003). Rational Approach to Risks [Dutch: Nuchter Omgaan Met Risico’s].

[5] RIVM (2017). A Scan of the Safety and Quality of Our Habitat [Dutch: Een Scan van de Veiligheid en Kwaliteit van Onze Leefomgeving].

[6] WRR (2008). Uncertain Safety [Dutch: Onzekere Veiligheid].

[7] Health Council of The Netherlands (2008). Prudent Precaution [Dutch: Voorzorg Met Rede].

[8] Health Council of The Netherlands (2012). Health Risks of Animal Husbandry [Dutch: Gezondheidsrisico’s Rond Veehouderijen].

[9] Briggs D.J. (2008). A framework for integrated environmental health impact assessment of systemic risks. Environ. Health.

[10] Leach M., Scoones I., Stirling A. (2010). Governing epidemics in an age of complexity: Narratives, politics and pathways to sustainability. Glob. Environ. Chang..

[11] Knol A.B., Briggs D.J., Lebret E. (2010). Assessment of complex environmental health problems: Framing the structures and structuring the frameworks. Sci. Total Environ..

[12] Slovic P. (2001). The risk game. J. Hazard. Mater..

[13] Adams J., Thompson M. (2002). Taking Account of Societal Concerns about Risk. Framing the Problem.

[14] Weiss C. (2003). Scientific Uncertainty and Science-Based Precaution. Int. Environ. Agreem..

[15] Douglas M. (1992). Risk and Blame, Essays in Cultural Theory.

[16] Douglas M., Wildavsky A. (1983). Risk and Culture, an Essay on the Selection of Technological and Environmental Dangers.

[17] Pidgeon N. (1998). Risk assessment, risk values and the social science programme: Why we do need risk perception research. Reliab. Eng. Syst. Saf..

[18] Lebret E. (2016). Integrated Environmental Health Impact Assessment for Risk Governance Purposes; Across What Do We Integrate?. Int. J. Environ. Res. Public Health.

[19] Alpaslan C.M., Green S.E., Mitroff I.I. (2009). Corporate Governance in the Context of Crises: Towards a Stakeholder Theory of Crisis Management. J. Cont. Crisis Manag..

[20] Seeger M.W. (2006). Best Practices in Crisis Communication: An Expert Panel Proces. J. Appl. Commun. Res..

[21] Venette S.J. (2007). Best practices in risk and crisis communication: Advice for food scientists and technologists. IUFoST Sci. Inf. Bull..

[22] Stirling A.C., Scoones I. (2009). From Risk Assessment to Knowledge Mapping: Science, Precaution, and Participation in Disease Ecology. Ecol. Soc..

[23] Tuler S., Webler T., Finson R. (2005). Competing perspectives on public involvement: Planning for risk characterization and risk communication about radiological contamination from a national laboratory. Health Risk Soc..

[24] Steelman T.A., Maguire L.A. (1999). Perspectives: Q-Methodology in National Forest Management. Policy Anal..

[25] Cuppen E., Breukers S., Hisschemoller M., Bergsma E. (2010). Q methodology to select participants for a stakeholder dialogue on energy options from biomass in The Netherlands. Ecol. Econ..

[26] HM Treasury (2005). Managing Risks to the Public: Appraisal Guidance.

[27] Ball D.J., Boehmer-Christiansen S. (2002). Understanding and Responding to Societal Concerns.

[28] Mansfield D. (2003). Gauging Societal Concerns. https://www.icheme.org/communities/subject_groups/safety%20and%20loss%20prevention/resources/hazards%20archive/~/media/Documents/Subject%20Groups/Safety_Loss_Prevention/Hazards%20Archive/XVII/XVII-Paper-02.pdf.

[29] Heederik D.J.J., Ijzermans C.J. (2011). Possible Effects of Intensive Animal Husbandry on the Health of Residents [Dutch: Mogelijke Effecten van Intensieve Veehouderij op de Gezondheid van Omwonenden].

[30] Kornalijnslijper J., Rahamat-Langendoen J., van Duynhoven Y. (2008). Public Health Aspects of Industrial Megafarms in The Netherlands Zoonoses and Antimicrobial Resistence [Dutch: Volksgezondheidsaspecten van Veehouderij Megabedrijven in Nederland Zoönosen en Antibioticumresistentie].

[31] Van Zeijts H., van Eerdt M., Farjon J. (2008). Environmental and Landscape Aspects of Megafarms among Industrial Farms [Dutch: Milieukundige en Landschappelijke Aspecten van Megabedrijven in de Intensieve Veehouderij].

[32] Health Council of The Netherlands (2011). Antibiotics in Animal Husbandry and Resistant Pathogens for Humans [Dutch: Antibiotica in de Veeteelt en Resistente Bacteriën bij Mensen].

[33] Geenen P.L., Koene M.G.J., Blaak H., Havelaar A.H., van de Giessen A.W. (2010). Riskprofile on Antimicrobial Resistence Transmissible from Food Animals to Humans.

[34] Dijkstra F.H., van der Hoek W., Wigers N., Schimmer B., Rietveld A., Wijkmans C.J., Vellema P., Schneeberger P.M. (2012). The 2007–2010 Q fever epidemic in The Netherlands: Characteristics of notified acute Q fever patients and the association with dairy goat farming. FEMS Immunol. Med. Microbiol..

[35] Kampschreur L., Delsing C.E., Groenwold R.H.H., Wegdam-Blans M.C.A., Bleeker-Rovers C.P., de Jager-Leclercq M.G.L., Hoepelman A.I.M., van Kasteren M.E., Buijs J., Renders N.H.M. (2014). Chronic Q fever in The Netherlands 5 years after the start of the Q fever epidemic: Results from the Dutch chronic Q fever database. J. Clin. Microbiol..

[36] Morroy G., Keijmel S.P., Delsing C.E., Bleijenberg G., Langendam M., Timen A., Bleeker-Rovers C.P. (2016). Fatigue following acute Q-fever: A systematic literature review. PLoS ONE.

[37] Tempelman C., Prins J., Koopmans C. (2011). Economic Consequences of the Q-Fever Outbreak [Dutch: Economische Gevolgen van de Uitbraak van Q-Koorts].

[38] Van Asseldonk M., Prins J., Bergevoet R. (2013). Economic assessment of Q fever in The Netherlands. Prev. Vet. Med..

[39] Dijk C.V. (2010). Policy Evaluation of the Q-Fever Outbreak 2005–2010 [Dutch: Van Verwerping tot Verheffing. Q-Koortsbeleid in Nederland 2005–2010].

[40] Ombudsman N. (2012). Het Spijt Me. Over Q-Koorts en de Menselijke Maat.

[41] Roodenrijs J.C.M., Kraaij-Dirkzwager M.M., van den Kerkhof J.H.T.C., Runhaar H.A.C. (2014). Risk governance for infectious diseases: Exploring the feasibility and added value of the IRGC-framework for Dutch infectious disease control. J. Risk Res..

[42] Lei L.E.I. (2011). Agricultural-Economic Report 2011 [Dutch: Landbouw-Economisch Bericht 2011].

[43] Paes M., Jans H., Van Santvoort M. (2011). Health Can Not Be Exchanged [Dutch: Gezondheid Is Geen Wisselgeld].

[44] (2010). Pledge for a Sustainable Animal Husbandry [Dutch: Pleidooi voor een Duurzame Veehouderij]. https://www.wanttoknow.nl/wp-content/uploads/pleidooi_voor_een_duurzame_veehouderij.pdf.

[45] Eijsackers H., Scholten M. (2010). About Careful Animal Husbandry (30 Essays) [Dutch: Over Zorgvuldige Veehouderij].

[46] Milieudefensie (2011). Factsheet What Is Wrong with Animal Factories? [Dutch: Wat Is er Mis Met Veefabrieken].

[47] Kanis E., Groen A.B.F., Greef K.H.D.E. (2003). Societal concerns about pork and pork production and their relationships to the production system. J. Agric. Environ. Ethics.

[48] Boogaard B., Oosting S., Bock B. (2008). Defining sustainability as a socio-cultural concept: Citizen panels visiting dairy farms in The Netherlands. Livest. Sci..

[49] Vanhonacker F., Verbeke W., Van Poucker E., Buijs S., Tuyttens F.A.M. (2009). Societal concern related to stocking density, pen size and group size in farm animal production. Livest. Sci..

[50] Frewer L. (2004). The public and effective risk communication. Toxicol. Lett..

[51] Van Der Giessen J.W.B., van De Giessen A.W., Braks M.A.H. (2010). Emerging Zoonoses: Early Warning and Surveillance in The Netherlands.

[52] Brugha R.V.Z. (2000). Stakeholder analysis: A review. Health Policy Plan..

[53] Bryson J. (2004). What to do when Stakeholders matter. Public Manag. Rev..

[54] Reed M.S., Graves A., Dandy N., Posthumus H., Hubacek K., Morris J., Prell C., Quimm C.H., Stringer L.C. (2009). Who’s in and why? A typology of stakeholder analysis methods for natural resource management. J. Environ. Manag..

[55] RLG (2008). Advice about Megafarm in Industrial Farming [Dutch: Het Megabedrijf Gewogen; Advies over Het Megabedrijf in de Intensieve veehouderij].

[56] Verhue D., Vieira V., Koenen B., van Kalmthout R. (2011). Opinions about Megafarms [Dutch: Opvattingen over Megastallen].

[57] Van Doorn C. (2011). All Meat Sustainable [Dutch: Al Het Vlees Duurzaam]. https://www.rijksoverheid.nl/documenten/rapporten/2011/11/23/al-het-vlees-duurzaam-de-doorbraak-naar-een-gezonde-veilige-en-gewaardeerde-veehouderij-in-2020.

[58] Bleeker H. (2011). Letter of the Underminister of Agriculture of The Netherlands to the Parliament of The Netherlands. Nr. 28973/48. https://zoek.officielebekendmakingen.nl/dossier/34359/kst-28973-48.html.

[59] Bleeker H., Schippers E. (2011). Letter of Minister of Health and Underminister of Agriculture of The Netherlands to the Parliament of The Netherlands. Nr 28973/67. https://zoek.officielebekendmakingen.nl/kst-28973-67.html.

[60] Parliament D. (2011). Transcript of Parliamentary Debate on Intensive Animal Husbandry.

[61] Corbin J., Strauss A. (1990). Grounded Theory Research: Procedures, Canons and Evaluative Criteria. Z. Soziol..

[62] VNG (2011). Onderzoek Toont Relatie Aan Tussen Intensieve Veehouderij en Gezondheid Omwonenden [Research Proofs Relationship Intensive Animal Husbandry and Health Residents]. http://www.vng.nl/onderwerpenindex/ruimte-en-wonen/nieuws/onderzoek-toont-relatie-aan-tussen-intensieve-veehouderij-en-gezondheid-omwonenden.

[63] Plattelandspost (2011). Geen Link Tussen Intensieve Veehouderij en Klachten [No Link between Intensive Animal Husbandry and Health Complaints]. http://www.plattelandspost.nl/835/geen-link-intensieve-veehouderij-en-klachten.

[64] Kayser M., Schlieker K., Spiller A. (2012). Gesellschaftlich keine Unterstützung. Fleischwirtschaft.

[65] Schmitt W. (2012). Charta für Landwirtschaft und Verbraucher—Künftige Herausforderungen für die Tierhaltung. Züchtungskunde.

[66] Brisson G., Mercier G., Godbout S., Lemay S.P. (2009). Élevage porcin et santé publique: Risque, controverse et violence non intentionnelle. Cah. Géogr. Qué..

[67] NCIFAP (2008). Putting Meat on the Table: Industrial Farm Animal Production in America. http://www.pewtrusts.org/en/research-and-analysis/reports/2008/04/29/putting-meat-on-the-table-industrial-farm-animal-production-in-america.

[68] Ball D.J., Boehmer-Christiansen S. (2007). Societal concerns and risk decisions. J. Hazard. Mater..

[69] Rowe G., Horlick-Jones T., Walls J., Poortinga W., Pidgeon N.F. (2008). Analysis of a normative framework for evaluating public engagement exercises: Reliability, validity, limitations. Public Underst. Sci..

[70] Alders H. (2011). From Mega to Better [Dutch: Van Mega naar Beter].

[71] KVHG Knowledge Platform on Animal Husbandry and Health 2013. http://www.kennisplatformveehouderij.nl/.

[72] Van Lieshout M. (2014). Framing the Scales and Scaling Frames. The Politics of Scale and Its Implications for the Governance of the Dutch Intensive Agriculture.

[73] Zon M.W. (2017). Talk in Action. https://www.zonmw.nl/nl/onderzoek-resultaten/gezondheidsbescherming/programmas/project-detail/non-alimentaire-zooenosen/talk-in-action-towards-a-constructive-dialogue-between-stakeholders-on-livestock-related-zoonoses/.

[74] Cousin M.-E., Siegrist M. (2010). The public’s knowledge of mobile communication and its influence on base station siting preferences. Health Risk Soc..

[75] Van Kleef E., Ueland O., Theodoridis G., Rowe G., Pfenning U., Houghton J., van Dijk H., Chryssochoidis G., Frewer L. (2009). Food risk management quality: Consumer evaluations of past and emerging food safety incidents. Health Risk Soc..

[76] Wolff J. (2006). Risk, Fear, Blame, Shame and the Regulation of Public Safety. Econ. Philos..

[77] Scholz R., Siegrist M. (2010). Low Risks, High Public Concern? The Cases of Persistent Organic Pollutants (POPs), Heavy Metals, and Nanotech Particles. Hum. Ecol. Risk Assess..

