# Supplementary material for: Rapid Assessment of Stakeholder Concerns about Public Health. An Introduction to a Fast and Inexpensive Approach Applied on Health Concerns about Intensive Animal Production Systems
Source: Int J Environ Res Public Health. 2017 Dec 11;14(12):1534. doi: 10.3390/ijerph14121534 (PMC5750952; doi:10.3390/ijerph14121534)
Supplement: Supplementary file 1 [file ijerph-14-01534-s001.pdf]

Table S1 Concerns and values involved in discussions about Intensive Animal Production Systems in the Netherlands (based on document analysis and interview transcripts as described in the article: Rapid assessment of stakeholder concerns about public health. An introduction to a fast and inexpensive approach applied on health concerns about intensive animal production systems).

(Public) Health	Infectious diseases (zoonoses)<br>Antibiotic resistant pathogens<br>Bio-aerosols (endotoxins)<br>Fine dust particles (also related to transportation of animals, food and materials on farms)<br>Odour<br>Gasses (ammoniac)<br>Reactions to chemical substances used in IAPS (incl. hormones and dioxine)<br>Obesitas/ too large consumption of animal fat & proteins (also stimulated by promotion by dairy industry)<br>Direct contact with living animals and nature has positive effects on human health (≠ IAPS)<br>Psycho-social complaints due to a) feelings of discomfort in crowded area (animals/humans) & b) loss of employment due to industrialization of agriculture<br>Relationship red meat and bowelcancer, gout
Animal Health	Infectious diseases<br>(↑ risk due to global movements of animals)<br>Genetic modification due to breeding for production causing physical complaints and lower immunity against diseases<br>Breeding economically driven (↑production), causing ↓animal health (↓ immunity, genetic narrowing)<br>Adapting animals for productionprocess (cutting mouth, sharp pieces)
Animal well-being	No opportunity for natural behaviour (little space, no possibility to go outside, piled in groups)<br>Scope of farm (space/ animal)<br>Instrinsic value of animal<br>Breeding economically driven (↑production), causing ↓well-being (problems giving birth, lack of muscle, genetic narrowing)<br>Transportation of live animals<br>Poor slaughtering conditions
Spatial planning; Landscape infrastructure; Livability rural area's	Loss of traditional rural landscape - replacement by large industrial complexes<br>Should Dutch rural areas further urbanize - industrialize or keep their traditional character?<br>Huge stable next door, devalues my view and my property<br>Infrastructure not ready for transportation of groundsources, animals, feeds and manure<br>Annoyance of industrial activities in close neighbourhood
Economic arguments	Agricultural sector (and its related industries) contributes to national income and employment<br>Discussion about desired 'free market principles' vs government intervention in agricultural sector<br>Autonomy farmers/entrepreneurs<br>('wish to earn a living in reasonable amount of hours/ wk')<br>Necessity for (economic) development/ growth farm, agricultural sector to survive<br>Lack of production control<br>Governmental subsidies unjust in economic market 'subsidized overproduction' (EU related)<br>Weak position of agricultural sector versus other industries? Maybe principle choice needed to invest in other industries than agricultural industry.<br>Who earns the profit, who pays the loss? (unfair)<br>"Low foodprice is being paid for by animals, environment, nature, ourselves and future generations"<br>Costs to control environmental and public health risks (not paid by those earning profit)
Environment	Manure overload (minerals; phosphate, nitrate, ammoniac, sulphate)

	Pollution of air, soil and water (manure, transport, industrialization, energy)<br>Climate change due to emission of CO_2, methane, N_2O<br>Loss of biodiversity (flora and fauna) due to environmental damage
Ecological footprint	Natural resources needed for IAPS are large and can be used better otherwise/ more proportional (see also sustainability & values)
Sustainability	Economic viability of agricultural sector<br>Societal acceptability of farms<br>Resource depletion (minerals, space, antibiotics)
Social values and emotions	Lack of acceptance of disease (fate) in Dutch modern society<br>Pretention to influence events [maakbaarheid] - blame of stakeholders involved in risk management if risk occurs.<br>The Netherlands is full - maximum total amount of animals and humans<br>Scope of farms in the NL - megafarms acceptable yes/ no?<br>Agriculture belongs to (traditions of) the Netherlands<br>Changing context (f.e. traditional milk cow farms change to large goat farms)<br>Harmony in rural communities under pressure<br>Alienation of agricultural practise, as no personal relation to animal husbandry (employment; dependency; involvement)<br>Alienation to animal husbandry (too romantic view ['Ot & Sienbeeld'], 'milk comes from the factory')<br>Livability and quality of life in rural area's (traffic, recreation, landscape, smell, industrialization, change in general, business, loss of traditions & community feeling).<br>'Loss of cow in the meadow'<br>"Must have feeling that what is happening inside is correct"<br>Many local risks, limited local benefits<br>Fear for (un)known (environmental) health problems<br>Slippery slope argument (now already bad, will become worse, if I don't fight it)<br>Loss of traditions (family farms)<br>Loss of natural approach to animal husbandry ("IAPS is unnatural" - "it has gone too far/ beyond acceptable limits")
Ethical values	Exploitation of animals<br>Time to change (like slavery; childlabour in earlier times)<br>Intrinsic value of animal (beneficence, non-maleficence, autonomy, justice) -> use of animal should be justified<br>Respect for life and nature (f.e. biblical values and stewardship [rentmeesterschap])<br>Using an animal as material ('verdingelijking′) instead of pet or living being (projection of human feelings = anthropromorf thinking?)<br>"We import the food, export the meat, keep the rubbish"<br>Killing healthy animals during outbreaks (including zoonosis), or not valuable for production<br>IAPS in the NL is not helping, possibly worsening, food security worldwide<br>Depletion of environmental resources worldwide (minerals, soja, clean H_2O, woodland,)<br>Use of natural resources to feed Dutch production animals causing social issues elsewhere (hunger, social unrest, loss of woodland)<br>Pluristic ignorance; unwillingness to see negative and immoral facts IAPS by many<br>"Food has become a 'throw-away-product' "
Socio-political	Whose interests prevail?<br>Who takes decisions and based on which criteria?<br>(Lack of) leadership in governance of risks/ societal concerns related to IAPS;<br>Too many stakeholders with large interests.<br>Which influence can general public have on IAPS (product and production process)? How effective is consumer influence through labelling and buying certain products? And how fair is the information provided on labels?
